# Development of retinopathy of prematurity

**Published:** 2017-11-11

**Authors:** Seema Qayyum

**Affiliations:** 1 Professor of Paediatric Ophthalmology: The Children's Hospital & Institute of Child Health, Lahore, Pakistan.

**Retinopathy of prematurity (ROP) is an ocular disorder which affects infants born before 34 weeks of gestation and/or with birth weight of less than 2000 grams. If not detected on time and appropriately managed, it can lead to irreversible blindness.**

## How does ROP develop?

**Figure 1:**
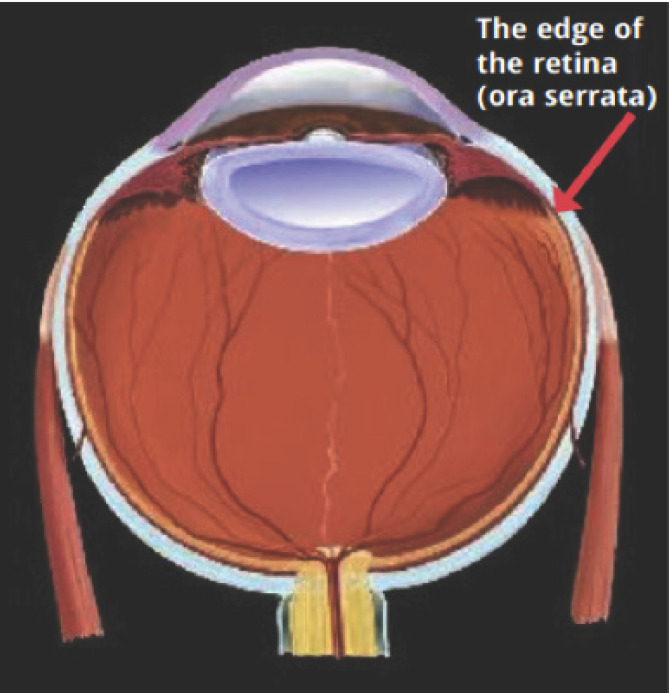
In a full term child, the retinal blood vessals are fully developed.

**Figure 2:**
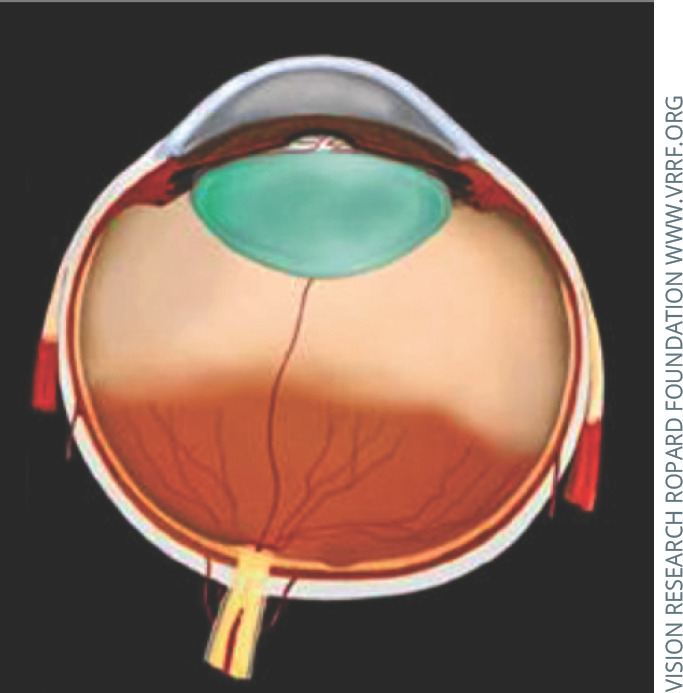
In a preterm infant, the retinal blood vessals are not fully developed.

The retinal blood vessels first appear between 15–18 weeks of gestation. These vessels grow outwards from the central part of the retina and extend towards the retinal periphery. The nasal part of the retina is fully vascularised by 36 weeks of gestation followed by the temporal retina which is completely vascularised between 36–40 weeks of gestation age (Figure 1). Following a premature birth, the growth of retinal blood vessels is halted and does not reach the periphery of retina (Figure 2).

**Figure 3:**
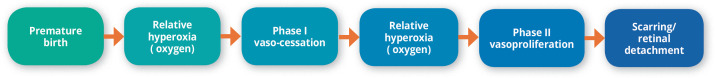
ROP can be divided into two phases

The development of ROP can be divided into two phases - an initial phase of delayed growth of retinal vessels followed by a second phase of retinal vessel proliferation (Figure 3).

### Phase I (Vaso-cessation): from birth to 30–32 weeks of gestation

At birth the lungs of an infant born preterm are immature placing him/her at a high risk of developing abnormally low level of oxygen in arterial blood.

To overcome this, the newborn infant is given supplemental oxygen in NICU. Prior to 32 weeks of gestation, the retina is very immature and the retinal metabolic demand is low. This excess oxygen creates retinal hyperoxia and oxygen toxicity, inhibiting the production of VEGF. This is followed by temporary stopping or stoppage of normal retinal growth, and constriction of new immature vessels. As a result, there is a reduction of blood supply to retinal tissue and shortage of oxygen needed for metabolism.

### Phase II (vaso-proliferation): after 30–32 weeks of gestational age

**Figure 4:**
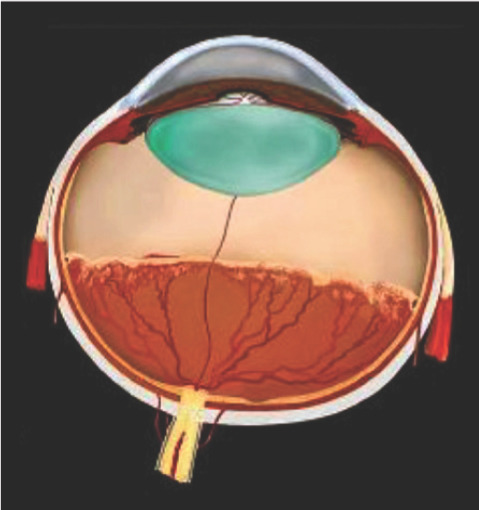
ROP in the junction of vascularised and non-vascularised retina

With increasing age of the preterm infant the retina matures. There is an increase in metabolic demand and oxygen consumption by the retina, creating a relative decrease in oxygen level. This promotes increase in the level of vascular endothelial growth factor (VEGF), triggering the formation of new blood vessels along the inner retinal surface. A demarcation ridge develops along the retina that separates the central vascularised retina from the peripheral avascular retina (Figure 4).

**Figure 5:**
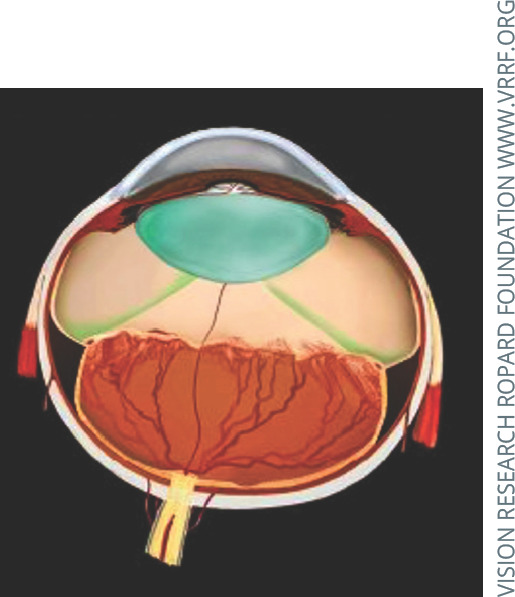
Advanced ROP with partial retinal detachment

The growth of retinal blood vessels at this stage may restart normally or may progress to significant ROP as seen by an abnormal growth of retinal vessels into the vitreous and over the surface of the retina. These new vessels are weak and underdeveloped failing to fulfill the oxygen demand of retinal tissue resulting in continuous growth of abnormal vessels. There is leakage of fluid or blood from these weak blood vessels. If not treated on time this can result in scarring or traction of the retina leading to retinal detachment and blindness (Figure 5).

